# The Key Site Variation and Immune Challenges in SARS-CoV-2 Evolution

**DOI:** 10.3390/vaccines11091472

**Published:** 2023-09-10

**Authors:** Ying Liu, Qing Ye

**Affiliations:** Department of ‘A’, Children’s Hospital, Zhejiang University School of Medicine, National Clinical Research Center for Child Health, Hangzhou 310052, China; 6521111@zju.edu.cn

**Keywords:** COVID-19, SARS-CoV-2, variation, key sites, immune challenge

## Abstract

Coronavirus disease 2019 (COVID-19) is a worldwide public health and economic threat, and virus variation amplifies the difficulty in epidemic prevention and control. The structure of severe acute respiratory syndrome coronavirus 2 (SARS-CoV-2) has been studied extensively and is now well defined. The S protein is the most distinguishing feature in terms of infection and immunity, mediating virus entrance and inducing neutralizing antibodies. The S protein and its essential components are also the most promising target to develop vaccines and antibody-based drugs. Therefore, the key site mutation in the S gene is of high interest. Among them, RBD, NTD, and furin cleavage sites are the most mutable regions with the most mutation sites and the most serious consequences for SARS-CoV-2 biological characteristics, including infectivity, pathogenicity, natural immunity, vaccine efficacy, and antibody therapeutics. We are also aware that this outbreak may not be the last. Therefore, in this narrative review, we summarized viral variation and prevalence condition, discussed specific amino acid replacement and associated immune challenges and attempted to sum up some prevention and control strategies by reviewing the literature on previously published research about SARS-CoV-2 variation to assist in clarifying the mutation pathway and consequences of SARS-CoV-2 for developing countermeasures against such viruses as soon as possible.

## 1. Introduction

Coronavirus disease 2019 (COVID-19) has spread rapidly, posing a severe threat to human health, economic development, and public health systems worldwide. COVID-19 has already lasted at least 3 years since 11 March 2020, when the World Health Organization (WHO) declared it a global pandemic. Currently, COVID-19 is drawing to a close, which has resulted in over 769 million confirmed cases and over 6.9 million deaths as of -August 2023. Over this period, the whole world expedited the search for effective responses and achieved notable success [1]. Both mRNA vaccines with greater than 90% effectiveness [2,3,4] and monoclonal antibodies (mAbs) alone or in combination therapy have been approved for emergency use [5,6,7]. However, these interventions target the initially severe acute respiratory syndrome coronavirus 2 (SARS-CoV-2). Viral mutations and accumulation lead to growing differences between the mutants and original strains and then a diminishing of antiviral medicine and vaccine effectiveness, posing a substantial barrier to pandemic prevention and control [8,9].

Viral genome mutation is a universal natural phenomenon in the course of evolution. These mutations in SARS-CoV-2 alter its structure and function, which, in turn, have an impact on host health and survival. The rapidly accumulated genomic data provide valuable resources for epidemiological studies in constant virus evolution during the pandemic. Therefore, the purpose of this review was to completely understand the viral structure, function, and cell entry of SARS-CoV-2, clarify its viral variation and prevalence condition, discuss specific amino acid replacement and its immune challenges, and attempt to summarize some prevention and control strategies in response to another resurgence. We hope that this work can contribute, in a modest way, to delineating the mutation pathway and consequences of SARS-CoV-2, to understand genomic evolution, and to shorten the development time of countermeasures for such viruses.

## 2. Structure, Function, and Cell Entry of SARS-CoV-2

SARS-CoV-2, an enveloped, single-stranded, positive-sense RNA virus, has a diameter of 100–160 nm and a genome length of 27–32 kb that belongs to the β coronavirus family. The SARS-CoV-2 genome consists of eleven genes, and five of them are confirmed to be particularly important, including the ORF1ab gene, S gene, E gene, M gene, and N gene. They each play a specific role in the viral cycle [10]. The ORF1ab gene (positions 266 to 21,555) occupies the first two-thirds of the genome, while the last third is occupied by the later reading frames, encoding four major structural proteins. Interspersed between these reading frames are the accessory protein-reading frames. The ORF1ab gene encodes the replicase polyprotein and then self-cleaves into 16 non-structural proteins (NSP1–NSP16), which are involved in RNA replication and transcription [11]. The S gene (positions 21,563 to 25,384) encodes the surface spike (S) glycoprotein, which interacts with the surface receptor angiotensin-converting enzyme 2 (ACE2) and enables the entry of the virus into host cells [12]. The E gene (positions 26,245 to 26,472) encodes the envelope (E) glycoprotein, a minor structural protein that is highly variable in different species, which is used to form pentameric proteolipid pores allowing for ionic transport after self-assembly in host cells, and is responsible for virus assembly and intracellular trafficking [13]. The M gene (positions 26,523 to 27,191) encodes the membrane (M) protein, the main structural protein of the envelope, playing a central role in providing the overall shape, virus assembly, budding, envelope formation, and pathogenesis stages by interacting with other proteins [14]. The N gene (positions 28,274 to 29,533) encodes the nucleocapsid (N) protein, which binds to the RNA genome in a continuous bead-on-a-string conformation and is used for protecting RNA and promoting RNA transcription, replication, and assembly [15].

**Figure 1:**
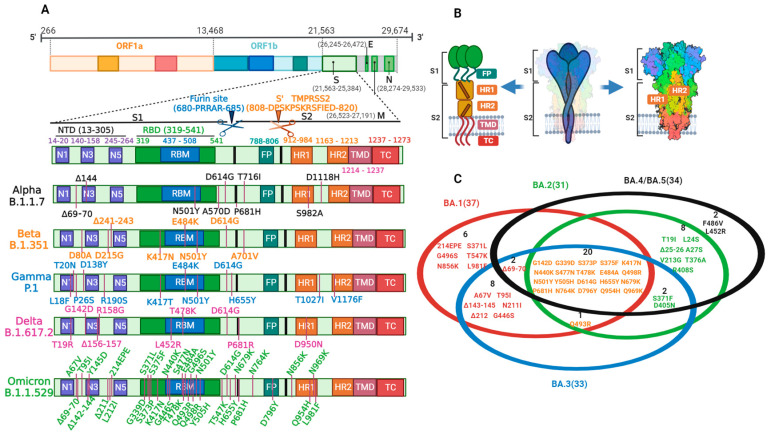
Variation mapping of the S protein from the prototype SARS-CoV-2 and its variants. (**A**) Architecture of the SARS-CoV-2 genome and variation mapping of the S protein from the prototype SARS-CoV-2 and its variants of concern (VOCs). (**B**) Schematic representation of the structure of the S protein. (**C**) Schematic representation of the variation mapping of the S protein in the subtype strains of omicron, including BA.1, BA.2, BA.3, BA.4, and BA.5.

The S protein is the most distinguishing feature of SARS-CoV-2 and is present on the virus surface as a homotrimer with remarkable motility and flexibility. The S protein mediates virus entrance and induces neutralizing antibodies during the immune response, which contains the most promising targets for vaccine- and antibody-based drug development. As shown in Figure 1A,B, each S monomer consists of an N-terminal S1 subunit and a C-terminal S2 subunit, as well as furin cleavage sites between them [16]. The S1 subunit forms the S head, while the S2 subunit forms the S stem, anchoring the S protein in the envelope. The S1 subunit involves an N-terminal domain (NTD, AA 14-305) and a receptor-binding domain (RBD, AA 319-541), and they are responsible for regulating the conformational changes of the S protein and for virus–receptor binding, respectively [17]. A short sequence within the RBD, called the receptor-binding motif (RBM, AA 437-508), interacts directly with ACE2. The S2 subunit is composed of a fusion peptide (FP, AA 788-806), a heptad repeat 1 (HR1, AA 912-984), a heptad repeat 2 (HR2, AA 1163-1213), a transmembrane region (TM, AA 1213-1237), and an intracellular domain (IC, AA 1237-1273); together, they mediate virus–cell membrane fusion [18]. Among them, HR1 interacts with HR2 to form the fusion-core structure 6-HB after virus–cell binding [19,20]. Furin cleavage sites (PRRAR) are specific amino acid sequences located at the S1/S2 boundary and are highly conserved among mammalian species. The cleavage and activation of these sites greatly boost virus entry efficiency. Viral cell entry is initiated by RBD-ACE2 binding, followed by the hydrolysis action of proteases, S1 shedding, S2 exposure and insertion into host cells, virus–cell membrane fusion, and viral genetic material release for replication [21]. These proteases accelerate efficient cell entry through cleaving and activating the receptor-attached S protein, which are mainly of two types and cleave the S protein at different sites: furin cleaves the S protein at the S1/S2 site to release S1, while transmembrane protease serine kinase 2 (TMPRSS2) cleaves it at the conserved site preceding the FP (S2′) to expose the internal hydrophobic FP in the S2 subunit that fuses the host and viral membranes together [22,23,24]. Progeny viruses are then exocytosed through secretory vesicles after completing translation and assembly. As soon as the progeny viruses have been released, they continue to infect other host cells [11].

## 3. SARS-CoV-2 Variation

Virus variation refers to the permanent alteration of genome nucleotide sequences for replication errors or other types of genetic material damage, thereby leading to changes in viral characteristics. Virus mutation is a natural process during replication and the foundation of evolution, meeting epidemiological rules. Compared with other microbes, viral infection with exponential proliferation tremendously increases the probability of mutation. The mutation sites of SARS-CoV-2 tend to focus on structural proteins, especially the S protein. This is because the structures and functions of non-structural proteins are highly conserved. Once mutations occur in these domains, viruses are highly likely to result in replication defects and then gradually phase out. However, as long as the basic skeleton remains unaffected, viruses with mutations in the structural protein are highly likely to be preserved. SARS-CoV-2 mutations occur randomly, but the host’s immune system and external environmental stress drive variation in a directional manner. Unfavorable variations will be gradually knocked out via natural selection, while favorable variations can be accumulated and sustained. For SARS-CoV-2, mutations are continuously shifting towards higher fitness through constantly enhancing its infectability and escape ability and decreasing pathogenicity. Ultimately, SARS-CoV-2 might coexist with humans for a long time. During this period, mutations with high virulence and lethality might occur by chance, but this phenomenon usually does not last long.

### 3.1. Variation Causes

For SARS-CoV-2, the large, infected population on a global scale offers tremendous opportunities for genetic variants. Compared with DNA viruses, SARS-CoV-2, as a single-stranded RNA virus, has poor replication fidelity, since there is no correction of its complementary strands, which is the core reason for the continuous emergence of new mutants. Therefore, a small probability of replication errors is inevitable, including deletions, mutations, and recombination. SARS-CoV-2 has relatively larger genomes among the RNA viruses, meaning that its occurrence probability of mutation is also comparatively greater. During viral replication, the limited error correction functionality of RNA-dependent RNA polymerase also greatly accelerates mutation. In addition, immunologic environmental changes in the host also contribute to virus variation, such as the emergence of drug-resistant mutants after the frequent use of mAbs [25]. In low-immunity populations, when viruses of different subspecies occasionally infect the same host, the replication system of host cells sometimes falsely exchanges the same gene segments of different viruses to form hybrid viruses. Such de novo variants are likely to exhibit some unpredictable properties after recombining two or more different types of virus. For example, the virus only infected one particular species before, but it can now infect humans following genetic recombination [26]. Furthermore, multiple outside environmental factors, such as temperature and UV irradiation, can also induce genetic mutations. Collectively, viral factors, the host immune environment, and the external environment all play important roles in SARS-CoV-2 variation.

### 3.2. Variation and Prevalence

SAR-CoV-2 is constantly mutating, and its infectivity has been mounting from the initial phase of the outbreak up to now. On 25 February 2021, the WHO published the definitions and working recommendations of the COVID-19 variants of concern (VOCs) and variants of interest (VOIs). Based on the amino acid changes in the S protein, five VOCs have been currently announced by the WHO, namely alpha (B.1.1.7), beta (B.1.351), gamma (P.1), delta (B.1.617.2), and omicron (B.1.1.529), as shown in Figure 1A. Alpha was first detected in the UK in September 2020 with a main epidemic peak time from January to July 2021, which had 10 amino acid variations in the S protein. Among them, Δ69–70 and Δ144 were located in the NTD, N501Y in the RBM, and P681H in the furin sites. Beta was first identified in South Africa in May 2020 with a main epidemic peak time from January to July 2021, which also had 10 amino acid variations in the S protein. Among these, D80A, D215G, and Δ242–Δ244 were located in the NTD, and K417N, E484K, and N501Y were located in the RBM. Gamma first appeared in Brazil in November 2020 with a main epidemic peak time from April to October 2021, which had 12 amino acid variations in the S protein. L18F, T20N, P26S, D138Y, and R190S were located in the NTD, and K417T, E484K, and N501Y were located in the RBM. Delta was first identified in India in October 2020 with a main epidemic peak time from July 2021 to January 2022, which had 10 amino acid variations in the S protein. Among them, T19R, G142D, Δ156–157, and R158G were located in the NTD, L452R and T478K in the RBM, and P681R in the furin sites. Omicron first appeared in South Africa in November 2021 with main epidemic peak times from November 2021 to now, which had 37 amino acid variations in the S protein. A67V, Δ69–70, T95I, G142D/Δ143–145, Δ211/L212I, and ins214EPE were located in the NTD, K417N, G446S, E484A, Q493R, G496S, Q498R, N501Y, and Y505H in the RBM, and P681H in the furin sites [27].

Omicron has rapidly swept across the globe since its initial characterization on 9 November 2021. On 26 November 2021, the WHO declared omicron the fifth VOC. In late December 2021, omicron quickly replaced delta as the dominant epidemic strain worldwide. Currently, omicron remains the predominant SARS-CoV-2 variant worldwide and has evolved into many subtype strains, including BA.1, BA.2, BA.3, BA.4, BA.5, and recombinant lineages. As shown in Figure 1C, the BA.1, BA.2, and BA.3 lineages have 37, 31, and 33 amino acid variations in the S protein, respectively. A total of twenty-one of these variations are common to all three lineages. Furthermore, 16 and 10 specific amino acid variations have been identified in the BA.1 and BA.2 lineages, respectively. BA.3, without specific variations, has ten shared variations with BA.1 and two shared variations with BA.2. BA.4 and BA.5 share the same variations in the S protein, which increase the additional variations (Δ69–70, F486V, L452R, and revertant Q493) on the basis of BA.2 mutations in the S protein.

Omicron’s diverse lineages have rapidly spread worldwide. From late December 2021 to late January 2022, the BA.1 lineage was the major prevalent strain. From late February to early June 2022, the BA.2 lineage overwhelmingly dominated during the global pandemic. Subsequently, the BA.4 and BA.5 lineages were identified in South Africa and then multiple countries, and rapidly replaced the dominance of BA.2. From July to November 2022, the BA.5 lineage was absolutely dominant in the global prevalence. From December to late January 2023, the dominant strain was the BQ.1 lineage, the sub-lineage of BA.5. In February 2023, XBB.1.5 quickly replaced BQ.1 as the global dominant epidemic strain. XBB is also an omicron subtype that seems to be generated via reassortment between BA.2.10.1 and BA.2.75, which can be further subdivided into different sub-strains, including XBB.1 and XBB.1.5. XBB.1.5 causes serious global concern, but compared with other omicron variants, its pathogenicity and clinical symptom differences have never been reported. No significant increase in confirmed cases, hospitalized cases, or mortality was found during the XBB.1.5 proportion increased period. Although XBB.1.5 remains dominant globally, its prevalence has been declining steadily. With the decline in testing and sequencing globally, the impact of emerging variants on disease severity remains unclear.

Since the end of December 2022, the number of confirmed cases and deaths from COVID-19 has rapidly declined over several months accompanied by a persistent increase in vaccination rates. Currently, the most severe phase has passed, and we human beings have entered a coexisting phase with SARS-CoV-2. It was announced by the WHO on 5 May 2023 that COVID-19 was no longer a public health emergency of international concern. On 5 December 2022, China announced the end of the COVID-19 outbreak and redefined SARS-CoV-2 as an “epidemic virus”. Meanwhile, all neighborhoods were no longer closed, and residents restored their freedom of action. After complete opening, China experienced an outbreak again from mid-December 2022 to early January 2023. However, this epidemic lasted a short time with low social hazard. Nowadays, SARS-CoV-2 tends to be a stable low infection state with mild symptoms. Occasionally, the situation of repeat positive cases arises, but symptom severity is relatively mild.

### 3.3. Variation and Immune Challenge of Key Sites

The mutation sites of SARS-CoV-2 tend to focus on structural proteins, but mutations in proteases within non-structural proteins should also be taken into consideration. Papain-like protease (PLpro) and main protease (Mpro or 3-CL) are two essential viral enzymes that process the polyproteins to generate NSP1–NSP16 during viral maturation [28], which are also promising targets for treating SARS-CoV-2 infections [29,30]. The P78L and K233Q mutations in the PLpro protein increased the death risk in infected individuals [31], while the V843F substitution promoted viral spread by reducing the affinity for ISG-15 [32]. M49I, L50F, Q189E, Q192T, N142L, Q189I, E166M, and E166V mutations in Mpro conferred strong nirmatrelvir resistance [33]. Further research on mutation mechanisms within non-structural proteins is still needed. Mutations in multiple structural proteins of SARS-CoV-2 have been demonstrated to entail a certain level of virulence in different ways, such as the S, M, and N proteins [34,35]. The S protein is the most critical component in terms of infection and the most variable component, so its variation is of high interest. Among them, the RBD, NTD, and furin cleavage sites are the most mutable regions, with the most mutation sites and the most serious consequences for SARS-CoV-2 biological characteristics, including natural immunity, vaccine efficacy, and antibody therapeutics. Each SAR-CoV-2 resurgence has been associated with key site variations in the above domains that enhance virus viability and transmissibility in distinct ways from wild-type (WT) strains, such as increasing receptor affinity, improving S protein stability, inducing cell–cell fusion, and facilitating immune escape. Other variations, such as D614G (the basis for other site variations), located outside the RBD, NTD, and furin cleavage sites, also have implications for virus fitness. Altogether, the key site variation modifies the severity of viral infections and is tightly linked to the initiation and progression of related diseases, which will be discussed in detail below.

**Figure 2:**
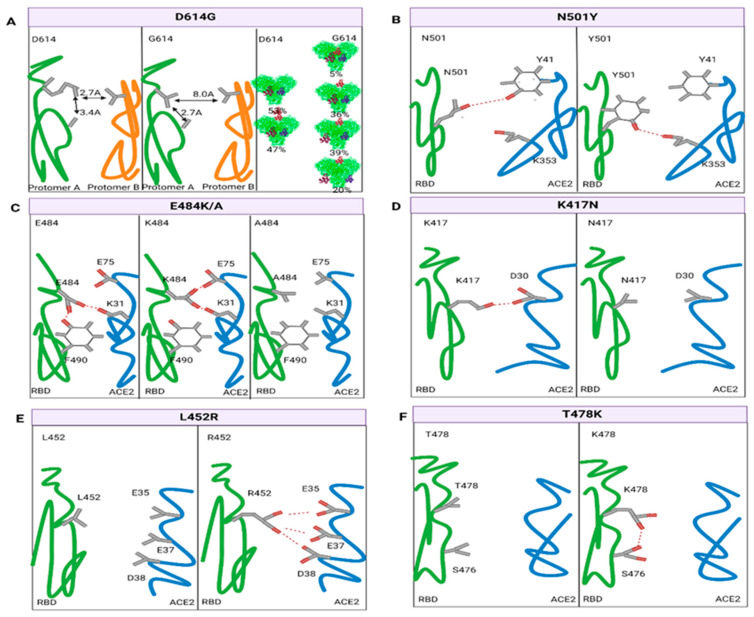
The mutant S protein RBD changes its binding affinity with hACE2 in comparison to the prototype SARS-CoV-2. (**A**) The D614G substitution decreases the binding affinity of RBD-ACE2, but it exhibits more efficient infection by making the RBD more open. (**B**) The N501Y substitution increases the binding affinity of RBD-ACE2 by forming pi-electron orbitals with Y41 and hydrogen bonds with K353 in ACE2. (**C**) The E484K substitution increases the binding affinity of RBD-ACE2 by forming a salt bridge between K484 and E75 in ACE2, while E484A decreases the binding affinity by abolishing weak contact with K31 in ACE2. (**D**) The K417N mutation attenuates the affinity for ACE2 by removing a interfacial salt bridge between K417 and D30 in ACE2. (**E**) The L452R substitution increases the binding affinity of RBD–ACE2 by increasing its electrostatic interactions with ACE2. Residue 452 is located in close proximity to a negatively charged patch of ACE2 residues (E35, E37, and D38). (**F**) The T478K increases S protein stability via intrachain interactions with S476. The red dashes represent hydrogen bonds, salt bridges, electrostatic interactions, or weak contacts between RBD and ACE2.

The D614G mutation refers to an amino acid change from aspartate (D) to glycine (G) at position 614, which is the first SARS-CoV-2 mutation to be discovered. All VOCs have the D614G mutation, which greatly facilitates virus adaptability. D614G virus infection resulted in a 3.7–8.2-fold higher infectivity than the WT virus in four susceptible cell lines. Moreover, compared to the WT virus, the D614G virus transmitted significantly faster and showed higher competitive fitness in hamsters. However, equivalent virus titers in the lungs and nasal turbinates, and similar histopathological lesion severity were demonstrated in both hACE2 mouse and hamster models [36]. There was no difference in mortality or clinical severity between patients infected with the 614G variant and the WT virus [37]. Fortunately, the D614G substitution did not significantly impact immune escape and shift the SARS-CoV-2 neutralization properties by detecting virus sensitivity to twenty-five convalescent human sera and six RBD mAbs [36,38]. Researchers have used cryo-EM to study the trimer of the S protein to find out how D614G increases infectivity. The D614G virus exhibits conformational changes due to the disruption of the interprotomer latch between D614 and T859 in S2. Figure 2A showed that these conformational changes were predominantly due to a dramatic ratio change in the S protein particles, from 53% being all closed and 47% one open for D614 to 5% all closed, 36% one open, 39% two open, and 20% three open for 614G [39]. It was physically impossible for ACE2 to bind to the RBD in its closed conformation [40]. The more open the RBD conformation was, the higher the overall affinity and infectivity of the virus. In spite of the fact that D614G does not affect S protein synthesis, processing, nor incorporation into host cells, its affinity for ACE2 is reduced due to a faster dissociation rate. However, the D614G variant is more efficient in terms of infection, replication, and competitive fitness in humans by opening up the RBD and then increasing lysis sensitivity [41].

#### 3.3.1. Variation in the RBD

The RBD binds strongly to ACE2 to initiate virus invasion, which is also a promising antigen target, as antibodies elicited against the RBD account for the majority of neutralizing antibodies (65–77%), which are often strongly neutralizing [42]. Amino acid alteration in the RBD, particularly in the RBM, changes the binding affinity of the S protein for ACE2 or for the primary target of neutralizing antibodies, and thus influences infectivity or causes immune evasion by reducing the protective effects from vaccination or prior infection [42,43]. Therefore, extra care must be taken regarding RBD mutations to prevent future spikes in infection and transmission.

The mutation N501Y converts the amino acid asparagine (N) into tyrosine (Y) at position 501, which is shared by alpha, beta, gamma, and omicron, and is also the only mutation of the RBD or the RBM in alpha. Studies have proven that the N501Y substitution was the determinant of the increased transmission of alpha, since only this substitution in the alpha S protein recapitulated the enhanced viral transmission [44]. With the help of a purified hACE2 ectodomain and different RBD models, studies confirmed that the N501Y substitution resulted in a 7-fold stronger binding to ACE2 than to the WT RBD [45]. The biolayer interferometry system also verified that the affinity was several hundred-fold higher in the N501Y-substituted RBD than in the WT RBD [44]. A pseudovirus with the N501Y mutation led to an 11.4–30.9-fold infection increase in 293T cells and a 23.5–37.8-fold increase in human airway organoids (HAO) relative to D614G alone [46]. The N501Y mutation imparted the cross-species transmission of SARS-CoV-2 to mice by enhancing receptor binding. Mice, as intermediate hosts, greatly accelerated SARS-CoV-2 transmission [47,48]. We next analyzed the mechanism of the N501Y mutation on hACE2 binding affinities and infectivity. The RBD residue N501 formed a hydrogen bond across the interface with hACE2 Y41, and a mutation into tyrosine resulted in this hydrogen bond loss. However, the aromatic Y501 side chain might stack onto hACE2 Y41 and form favorable van der Waals interactions using its pi-electron orbitals (Figure 2B). Additionally, the Y501 hydroxyl group formed a hydrogen bond and a hydrophobic interaction with hACE2 K353 [45]. These factors collectively contribute to the stronger binding affinity and infectivity of the N501Y variant. Fortunately, the N501Y variant does not gain the ability to evade immune recognition and cannot diminish vaccine efficacy. The N501Y mutation dramatically reduced the neutralization of several mAbs; however, polyclonal antibodies from infected individuals remained active against it [49]. The BNT162b2-vaccinated population possessed an equivalent resistance ability to the alpha variant [50]. Overall, N501Y increases viral fitness for replication and transmission by enhancing S-ACE2 interactions, which was also the leading cause of the alpha variant pandemic worldwide in early 2021.

The 484 site variation occurs in the RBM of beta, gamma, and omicron. Beta and gamma have a substitution of glutamate (E) with lysine (K), while omicron displays a substitution with alanine (A). Studies have shown that the E484K mutation slightly enhanced RBD-ACE2 binding [51], but E484A decreased the binding affinity [52]. As shown in Figure 2C, E484 formed a hydrogen bond with F490, which attached the loop (residues 477–486) to the remainder of the RBD. The mutation abolished this hydrogen bond and allowed the loop to separate from the remainder of the RBD. However, due to electrostatic attraction, K484 was gradually moved towards E75 in ACE2 to form a salt bridge [53]. When it was mutated to A484, the side chain was too short to interact with hACE2, resulting in a decreased affinity [52]. Both E484K and E484A helped to stimulate the severe immune escape of the virus [54]. Four variants (E484A, E484D, E484G, and E484K) all exhibited broad resistance to each of the human convalescent sera they tested and produced varying degrees of resistance across the entire panel of antibodies (19 mAbs against the RBD). E484A exhibited the highest resistance, followed by E484K and E484G [55]. Many studies have demonstrated that neutralization by some convalescence sera of E484 mutation was reduced >10-fold [56,57,58]. The E484K mutation also reduced neutralization via post-vaccination sera. Experiments indicated that the E484K mutation reduced the neutralization potency of post-vaccination sera (mRNA-1273 or BNT162b2) against SARS-CoV-2 pseudo-typed viruses at least 10-fold [59]. The neutralization efficiency of sera from individuals who received two doses of BNT162b2 was lower with the E484K mutation versus USA-WA1/2020 by 3.4-fold [57]. Another study revealed that there was a significant reduction in the neutralizing activity of post-mRNA-1273 vaccination sera in the presence of the E484K mutation after comparing its neutralizing activity against the alpha with that against the alpha + E484K [60]. The mean fold decline in neutralizing activity for alpha + E484K was 6.7, compared with 1.9 for alpha relative to the WT [61]. Similar results were later reported with post-BNT162b2 vaccination sera. A large population-based study in Qatar suggested that the estimated effectiveness of BNT162b2 against the alpha variant was 89.5% at 14 or more days after the second dose, while the effectiveness against the beta variant was 75.0% [62]. In addition, the E484K mutation was refractory to neutralization by most mAbs against both the NTD and RBM [63]. However, several mAbs and combination therapy still retain their activity against E484K [64]. Taken together, the E484K mutation not only slightly enhances RBD-ACE2 binding, but also significantly increases immune escape by reducing the effectiveness of convalescent plasma, vaccines against SARS-CoV-2, and therapeutic mAbs.

The 417 site variation occurs in beta, gamma and omicron. Beta and omicron have a substitution of lysine (K) with asparagine (N), while gamma displays a substitution with threonine (T). Despite their different biochemical characteristics and binding patterns, both give similar attributes to the variants [65]. As shown in Figure 2D, the K417N/T mutation reduced affinity for the receptor by removing one interfacial salt bridge between K417 in the RBD and D30 in ACE2 [53,66]. Consequently, the variant with this single mutation has a lower infection capacity than the WT virus. However, this mutation has not been observed alone in any known SARS-CoV-2 variant so far, which is frequently accompanied by the N501Y and E484K mutations. The improved RBD-ACE2 affinity from both N501Y and E484K was sufficient to compensate for the loss caused by the K417N/T mutation, and the combined effect yielded a stronger interaction with ACE2 [53]. The K417N mutation is also associated with the potential to escape neutralization from various mAbs, convalescent sera, and post-vaccination sera. This mutation abolished a buried interfacial salt bridge between K417 in the RBD and D104 in the therapeutic neutralizing antibody CB6 to reduce affinity by 9.59 kcal/mol, which facilitated the variant to efficiently elude such antibodies [53]. A study found that the neutralization of four and five antibodies out of eighteen tested antibodies isolated from COVID-19 patients or humanized mice was abolished by K417N and E484K, respectively. It is striking to note that the binding and neutralization capacity of all six highly potent IGHV3-53/3-66 antibodies were significantly reduced in the presence of either K417N or E484K mutations [67]. Moreover, five of the seventeen antibodies from post-vaccination sera (mRNA-1273 or BNT162b2) were at least 10-fold less potent against K417N [59]. Overall, the K417N mutation of SARS-CoV-2 seems to sacrifice its ACE2 binding affinity to survive antibody attacks.

L452R has a leucine-to-arginine substitution at position 452, which is specific to the delta of the VOCs. Analysis of RBD–ACE2 complexes revealed that the L452R residue was positioned in the RBM, but that it did not directly contact the receptor [68]. Structural analysis and in silico mutagenesis suggested that the L452R substitution enhanced RBD-ACE2 affinity by promoting electrostatic complementarity between R452 and the negatively charged patch of ACE2 residues (E35, E37, and D38) (Figure 2E) [69,70]. To prove the separate effect of L452R on viral entry, pseudoviruses carrying D614G-L452R or D614G alone were generated. Increased entry of D614G-L452R pseudoviruses was observed compared to D614G alone, with a 6.7–22.5-fold increase in 293T cells and a 5.8–14.7-fold increase in HAOs [71]. This mutation would facilitate virus penetration, giving it greater infectivity and transmissibility. Moreover, L452R has been proven to have an impact on immunity. It had been shown that the L452R mutation inhibited HLA-restricted cells from recognizing antigens and compromised CD8+ cell activation, which helped the variant escape HLA-restricted cellular immunity and further exacerbated infection progression [69,72]. In addition, studies have shown that L452R endowed the mutant with moderate resistance to antibodies elicited by previous infection or vaccination [71,73]. This mutation reduced neutralizing activity in 14 of 34 RBD-specific mAbs, and 10 of them experienced a decline of 10-fold or more in their neutralization potency [74]. In brief, the L452R mutation significantly enhances the infectivity of the virus and promotes immune escape by increasing affinity to hACE2, influencing cellular immunity, and reducing the neutralization of post-vaccination and convalescent sera.

The T478K variation is a threonine-to-lysine substitution shared by delta and omicron. The T478K residue was also positioned in the RBM, but it did not directly contact the receptor. K478 increased S protein stability via intrachain interactions with S476 (Figure 2F) [75]. According to an in silico molecular dynamics study, the T478K mutation replacing a non-charged threonine with a positive lysine significantly altered the electrostatic surface of the S protein and then enhanced the ACE2 interaction, which could be increased if combined with other S mutations, such as D614G, P681H, and L452R [76]. Nevertheless, deep mutational scanning of the RBD revealed that the T478K mutation did not significantly affect hACE2 binding [51]. In spite of these conflicting results, later studies confirmed that the T478K mutation enhanced infectivity and immune escape, and decreased susceptibility to mRNA vaccines [76,77,78].

There are 11 additional mutations newly discovered in the omicron RBD, including G339D, S371L, S373P, S375F, N440K, G446S, S477N, Q493R, G496S, Q498R, and Y505H. The omicron RBD binds to hACE2 with similar affinity to the WT RBD due to multiple mutations compensating for each other. Among them, the G446S, G496S, and Y505H substitutions decreased RBD-ACE2 affinity, whereas S477N, Q493R, and Q498R compensatively increased the affinity [52]. The S371L, S373P, and S375F mutation combination provided a substantial advantage for stabilizing the RBD-down interface [79], while the 498R and N501Y combination has been shown to enhance ACE2 binding above that of N501Y alone in an epistatic manner [80]. Moreover, the rapid spread of N501Y in the population increased the emergence likelihood of Q498R. Overall, the infectivity of omicron increased by 2–3-fold compared with delta [81]. It was observed that RBD mutations of previous variants were restricted to sites within class 1 and 2 antibody epitopes, while omicron broadened antibody escape. Class 3 antibody escape was strongly associated with mutations N440K, G446S, G496S, and Q498R. An additional set of mutations affected the majority of class 4 antibodies and select class 3 antibodies via indirect mechanisms, including G339D, S371L, S373P, and S375F [79]. Multiple synergetic mutations resulted in more than 85% of neutralizing antibodies escaping via omicron. The neutralization capacity of sera immunized with a double mRNA1273 vaccine and a BNT162b2-boosted vaccine for omicron was approximately 20- and 22.7-fold lower than that for the WT viruses, respectively [82]. With regard to neutralizing antibody-based drugs, the neutralization potency of the majority of them was greatly undermined by omicron.

Collectively, numerous variations, including G446S, L452R, T478K, E484K, G496S, N501Y, Y505H, and D614G, were associated with an increased ability to penetrate and infect host cells, with the variations L452R and N501Y exhibiting greater infectivity. Compared with the L452R mutation, the infectivity increase in the variant with the N501Y mutation was slightly higher [71]. It was demonstrated that substitutions at residues T345, R346, K417, K444, G446, N450, L452, S477, T478, E484, F486, and P499 were each associated with resistance to more than one mAb, with three of them (K417, S477, and E484) exhibiting broad resistance and E484 substitutions showing the highest resistance [55]. It was found that E484A, E484D, E484G, and E484K all displayed broad resistance to the convalescent sera from humans. E484A exhibited the highest resistance, followed by E484K and E484G. The triple mutants (K417N/T-E484K-N501Y) seriously threaten the efficacy of the already developed vaccines. Moreover, the lethality of the triple mutants was greater than that of the N501Y variant due to interprotein contact, specifically electrostatic contact, while N501Y was comparable to the WT virus [65].

#### 3.3.2. Variation in Furin Cleavage Sites

The furin cleavage sites played an essential role in invading host cells, and due to its existence, cell entry was more efficient in SARS-CoV-2 than in SARS-CoV [83]. To gain an insight into its physiological roles, a mutant of SARS-CoV-2 lacking the furin sites was constructed and used, and it was found that it reduced replication in the human respiratory cell lines and attenuated pathogenesis in both the hamster and K18-hACE2 transgenic mouse models [84]. Based on a pseudovirus infection assay, SARS-CoV-2 infection in humans was strongly reduced via a protease inhibitor or siRNA targeting the furin sites. This information demonstrated a critical role of furin sites in infection and reminded its importance in mutation.

The 681 site mutation occurs in beta, gamma, and omicron. Alpha and omicron have a proline-to-histidine substitution (P681H), while delta displays a proline-to-arginine substitution (P681R). The cleavage activity of the furin sites is influenced by pH, and a low pH poses an inhibitory effect, whereas P681H and P681R mutations both cause the surface to become more alkaline to improve the cleavage effect by providing additional basic residues. Compared with the WT or alpha strain, the P681H mutation slightly increased S1/S2 cleavage, but this did not significantly impact viral entry or cell-to-cell spread via functional and cell-to-cell fusion assays [85]. The P681H mutation escaped IFITM restriction and was necessary for type I interferon resistance, whereas mutation reversion was sufficient to restore its prior sensitivity [86]. Notably, compared with the D614G mutant, the D614G-P681R mutant significantly facilitated furin-mediated S cleavage by increasing the cleaved S2 subunit level and markedly enhanced cell–cell fusion by forming larger syncytia [87]. According to a pseudovirus neutralization assay, the D614G-P681R mutant showed moderate resistance (approximately 1.5 times) to several important mAbs against the RBD [87]. In addition, the BNT162b2 vaccination sera showed a significant reduction in neutralizing antibody titer against the D614G-P681R virus. To gain an insight into the influence of the P681R point mutation and the difference between P681H and P681R, researchers constructed alpha and delta variants with a single genetic background via reverse genetics. Their results indicated that delta had a greater replication fitness (1.7–7 times) than alpha in a competition assay in respiratory models of SARS-CoV-2 infection in vitro, including human lung adenocarcinoma Calu-3 cells and human airway epithelial (HAE) cultures. After exchanging the S of alpha and delta, a competition assay confirmed that S drove the improved replication of delta over alpha. Reverting P681R into WT P681 significantly reduced delta replication to a level lower than that of alpha [88]. Collectively, the P681H mutation slightly increases S cleavage, but does not significantly impact viral entry or cell–cell fusion, whereas the P681R mutant significantly facilitates furin-mediated S cleavage, markedly enhances cell–cell fusion, and endows a moderate immune escape ability.

#### 3.3.3. Variation in the NTD

NTDs also play important roles in protective immunity, viral evolution, and vaccine design, and mutations in NTDs are frequently detected in variants. The NTD is also an important identification region of mAbs, and NTD mAbs accounted for 5–20% of S-specific mAbs cloned from memory B cells isolated from the PBMCs of COVID-19 individuals [89]. Beyond neutralization, NTD mAbs mediated an array of additional antiviral functions via Fc-mediated effects or functions. Indeed, mutations in the NTD are equally concerning, and strengthened monitoring is imperative.

The Δ69–70 mutation is the most prominent mutation in the NTD, which is characterized by two amino acid deletions at positions 69/70 (H69/V70) and arises in multiple lineages, such as alpha and omicron. Compared with the WT virus, the Δ69–70 mutation led to a 2-fold increase in infectivity in a range of target cells, including HeLa cervical epithelial cells, A549 lung cells, and Calu-3 lung adenocarcinoma cells, which was associated with increased cleaved S incorporation [90]. In addition, the Δ69–70 mutation potentially provided an additional rapid route for virus dissemination by mediating faster syncytium formation among neighboring cells, which had been demonstrated to mediate faster fusion kinetics than the WT strain bearing D614G by forming syncytium, and this enhancement was abrogated via reinsertion of the H69 and V70 residues [90]. Although Δ69–70 allosterically alters the NTD’s conformation, it is not a main neutralizing antibody escape mechanism. Researchers have only observed a slight decrease in the susceptibility and binding of convalescent sera and 12 NTD mAbs [61,90]. Moreover, all neutralizing NTD mAbs bound efficiently to variants carrying Δ69–70, indicating that targeting mAbs would not be affected by these mutations [42]. Δ69–70 had a great impact on infection diagnosis, leading to S gene dropout and producing a negative result [91]. Multiple mutations in the NTD are related to a reduced or abrogated recognition of some NTD-specific mAbs and thus lead to immune escape. L18F, D80A, D253G/Y, or S255F variants only abrogated the binding of S2L28 to the NTD. Conversely, Δ144 abrogated binding to S2M28, S2X28, S2X333, and 4A8 but not S2L28. The H146Y mutant reduced the binding of S2M28, S2X28, and, in particular, 4A8 [42].

## 4. Prevention and Control Strategies

SARS-CoV-2 variation greatly increases the difficulty of epidemic prevention and control by increasing viral infectivity, pathogenicity, and immune escape. So, how can this conundrum be prevented and controlled? In order to win valuable time for vaccine or antiviral drug design, the in vitro evolution method should be established and improved to predict early and discover high affinity or pathogenic mutations. There have been studies reporting the application of an in vitro evolution method (an enhanced yeast surface display protocol) to select for the higher affinity binding of the RBD to the host cell receptor ACE2, and it was surprising to observe that natural SARS-CoV-2 selection followed the same path [80]. In addition, more comprehensive and sensitive methods than qRT-PCR are needed to help us monitor, track, and discover new mutations and variants timely, such as high-throughput sequencing technologies. Nucleic acid vaccines for human use spawned by COVID-19 are potent weapons for controlling new variants, since they make it possible to rapidly and inexpensively prepare targeted vaccines based on mutational antigenic epitopes on a large scale. Moreover, nucleic acid vaccines could elicit both potent and long-term humoral and cellular immune responses [92]. However, the development of this vaccine is still in its infancy, so further research and upgrades are needed to ensure its long-term safety. Before the new vaccines are available, the booster vaccination is considered a simple and the most cost-effective method for the fast spread of variant control, particularly the heterologous prime-boost immunization [93,94]. The Pfizer study highlighted a significant immune response among humans after a third BNT162b2 vaccine dose, although two-dose vaccinees showed a notable decrease in neutralizing activity against omicron. Almost all two-dose vaccine recipients were undetectable for omicron neutralization. However, individuals boosted with mRNA vaccines were able to potently neutralize omicron [95]. The combined application of multiple antibodies or antiviral drugs can also partially ameliorate the immune escape of variants via synergistical enhancement. We should also be aware that this outbreak may not be the last epidemic, and that it is the inevitable result and the direction for evolution. In order to minimize universal health risk, this requires us to strengthen self-protection awareness, strengthen health infrastructure construction, and improve the health regime.

## 5. Conclusions

SARS-CoV-2, a novel coronavirus, can infect host cells with a high efficiency due to the coexistence of both proteases, which has led to the mortality of millions of people and economic disruption worldwide. The continuous emergence of new variants further exacerbate the difficulty in epidemic prevention and control. Virus variation is a widespread biological property. Although mutations occurring in critical domains do not necessarily impact viral characteristics due to the degeneracy of the genetic code and similarity of amino acids, some incidental and non-oriented mutations still result in virulence and lethality spurt by increasing viral infectivity and immune escape. Accordingly, the SARS-CoV-2 pandemic and variation caught the world off guard, which requires us to strengthen surveillance and prediction capabilities, expedite variant vaccine development, and further improve the health infrastructure construction and regime.

## Data Availability

The data that support the findings of this study are available from the corresponding author upon reasonable request.
